# Women's views on lifestyle changes to reduce the risk of developing Type 2 diabetes after gestational diabetes: a systematic review, qualitative synthesis and recommendations for practice

**DOI:** 10.1111/dme.13926

**Published:** 2019-03-04

**Authors:** R. A. Dennison, R. J. Ward, S. J. Griffin, J. A. Usher‐Smith

**Affiliations:** ^1^ Primary Care Unit Department of Public Health and Primary Care University of Cambridge Cambridge UK; ^2^ MRC Epidemiology Unit University of Cambridge Cambridge UK

## Abstract

**Aims:**

After gestational diabetes, many women exhibit behaviours that increase their risk of developing Type 2 diabetes. We aimed to systematically synthesize the literature that focuses on the views of women with a history of gestational diabetes on reducing their risk of developing diabetes postpartum through lifestyle and behaviour changes.

**Methods:**

We identified qualitative studies that examined the views of women with a history of gestational diabetes towards healthy eating and physical activity, Type 2 diabetes risk management or their experience of a diabetes prevention programme, and conducted a thematic synthesis to develop descriptive and then analytical themes. We also evaluated the quality of each study and the confidence that we had in our findings.

**Results:**

We included 21 articles after screening 23 160 citations and 129 full texts. We identified six themes of interacting influences on postpartum behaviour: role as mother and priorities; social support; demands of life; personal preferences and experiences; risk perception and information; and finances and resources (plus preferred format of interventions). These factors inhibited many women from addressing their own health, while they motivated others to persevere. We also developed 20 recommendations, most with high or moderate confidence, for effective promotion of healthy lifestyles in this population.

**Conclusions:**

Many factors hinder healthy lifestyles after gestational diabetes, yet how women interpret them can motivate or prevent changes that reduce diabetes risk. As our recommendations emphasize, women's experiences and needs should be considered when designing strategies to promote healthier lifestyles in this population.

## What's new?

- After having had gestational diabetes, many women do not adopt healthy lifestyles that would reduce their risk of developing Type 2 diabetes.
- We found, in summary, that women identified themselves primarily as mothers who prioritized their family above themselves, and needed resources, time, energy, information and support to encourage healthy diets and levels of activity.
- Based on these findings, we developed 20 recommendations for effectively promoting healthy lifestyle in this population. These recommendations highlight the need for interventions to be centred on women's needs and experiences.

**What's new?**

- After having had gestational diabetes, many women do not adopt healthy lifestyles that would reduce their risk of developing Type 2 diabetes.
- We found, in summary, that women identified themselves primarily as mothers who prioritized their family above themselves, and needed resources, time, energy, information and support to encourage healthy diets and levels of activity.
- Based on these findings, we developed 20 recommendations for effectively promoting healthy lifestyle in this population. These recommendations highlight the need for interventions to be centred on women's needs and experiences.

## Introduction

Gestational diabetes (GDM) is a common disorder of pregnancy and the single most important risk factor for the development of Type 2 diabetes 1, 2, 3. It is defined as diabetes with an onset or first diagnosis during pregnancy and increases the risk of adverse pregnancy outcomes for both mother and baby 4. Many find it distressing, with the shock of diagnosis followed by self‐blame and anxiety for the unborn baby or, for some, motivation to take control of their health during pregnancy such as managing GDM by following advised lifestyle changes 5, 6.

Glucose control typically returns to normal after delivery and maternal care tends to focus on regular screening for diabetes 4, 7. Postpartum health behaviours (specifically healthy diet and physical activity) are strongly associated with diabetes risk; however, most women do not attempt behaviour change, instead maintaining lifestyles that increase their risk 8. In the UK, women are managed according to the guidelines for preventing Type 2 diabetes 4. These include referral to weight‐loss or exercise programmes 9. Such programmes were developed for the general population, which tends to be older and not to have young families. Current evidence also shows that interventions to prevent Type 2 diabetes after GDM can have positive, but sometimes limited, effects if engagement is poor 10, 11, 12. Notably, there was ~50% lower incidence of diabetes after GDM in the Diabetes Prevention Programme (DPP) after intensive lifestyle and metformin intervention compared with placebo after 3 years 13, indicating potential benefits of behavioural interventions on diabetes outcomes.

Previous qualitative or mixed methods reviews have explored women's postpartum views on reducing diabetes risk as part of broader investigations into their experience of GDM 6, 14, 15, 16. A wide variety of views and determinants have been presented, including positive attitudes towards behaviour change, particularly when it is understood to reduce diabetes risk and when women have support and self‐efficacy for change. Changes can be prevented by lack of information, support, time and help with childcare. To date, however, no comprehensive review has focused on postpartum lifestyle.

We have systematically synthesized the literature reporting the views of women with a history of GDM on reducing their risk of developing diabetes, including women participating in interventions. These findings help to identify gaps in our understanding of the acceptability, feasibility and practicality of intervening postpartum and will subsequently inform the development or tailoring of effective approaches for this easily identifiable, high‐risk population.

## Methods

Details of the review protocol were registered on PROSPERO (available at https://www.crd.york.ac.uk/prospero/display_record.php?RecordID=82049).

### Search strategy

We searched MEDLINE, Embase, PsychINFO, CINAHL and the Cochrane Library electronic databases in September 2017 as part of a group of literature reviews concerning GDM using the search strategy shown in Table S1. No language or other restrictions were applied. Reference lists of included studies were screened for citations not identified by this search.

### Study selection

Our predefined selection criteria included studies published in peer‐reviewed journals that examined women's experiences of healthy eating and physical activity after GDM, views on diabetes risk management, or experience of a diabetes prevention programme. All qualitative methods were eligible, including mixed methods. Views of healthcare providers and about postpartum diabetes screening were excluded in order to focus on lifestyle.

After deduplication, all titles and abstracts were assessed against the selection criteria by R.D. or R.W. Both authors reviewed ~10% of the citations to ensure agreement. Any differences were discussed, and the selection criteria were refined and elaborated in conjunction with the other authors so that they could be applied consistently. Full‐text articles were then acquired and rechecked against these criteria by R.D. J.U‐S. reviewed all included articles as well as those excluded for reasons other than article type, and agreed with the classification.

### Quality assessment

With discussion with the other authors, R.D. assessed the quality of each study's qualitative findings against the Critical Appraisal Skills Programmes (CASP) checklist for qualitative research 17. No studies were excluded based on quality.

### Qualitative synthesis

**Figure 1:**
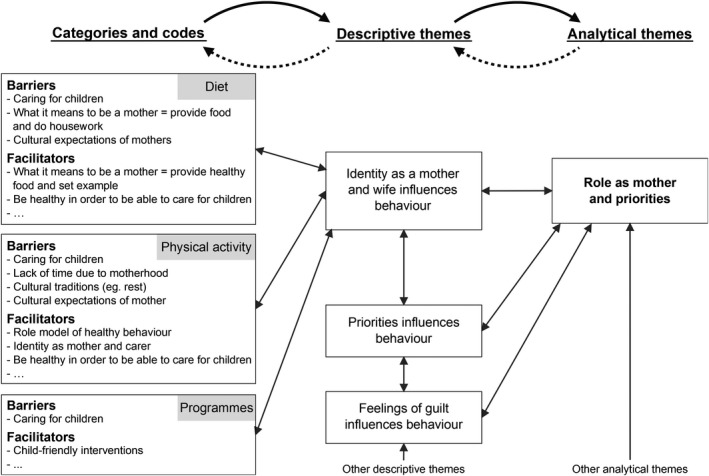
Example of development of the analytical theme ‘Role as mother and priorities’ within the thematic synthesis. Actual and anticipated barriers and facilitators were combined in this diagram and not all codes were presented for simplicity.

Data were defined as text or tables labelled as ‘Results’ (or equivalent) that arose from qualitative methods. Data were analysed using thematic synthesis, as described by Thomas and Harden 18, with the aid of Nvivo 11. After carefully reading and re‐reading each primary study, we coded the findings, organized these codes into related areas to develop descriptive themes and then developed analytical themes. The first stage was completed in two steps: firstly, data were categorized into anticipated or experienced barriers and facilitators to healthy diet, physical activity and participation in an intervention programme, alongside other information such as perception of diabetes risk. Secondly, codes were developed within these categories. R.D. extracted and coded the data, with J.U‐S. independently coding a subset of papers at multiple stages to check consistency. In the next stage, concepts were translated from one study and category to another by making summaries and comparisons, and new concepts were developed as shown in Fig 1. Themes were discussed with all authors throughout.

Illustrative quotations from the original studies are reported alongside analytical themes to allow appreciation of the primary data. We considered our perspectives on the findings as clinical or non‐clinical researchers in the UK throughout this process. S.G. and J.U‐S. are general practitioners with qualitative research experience, R.W. is an academic general practice registrar and R.D. has undertaken postgraduate training in public health and completed this research as part of her doctoral studies.

### Recommendations for promoting behaviour change

We developed 20 recommendations for promoting healthy postpartum lifestyle based on our results, and considered which behaviour change techniques could be used to implement them in line with the behaviour change technique taxonomy (v.1) 19. Our confidence in each recommendation was assessed using the Grading of Recommendations Assessment, Development and Evaluation‐Confidence in Evidence from Reviews of Qualitative Research (GRADE‐CERQual) approach 20 and discussed in order to inform the final interpretation.

## Results

**Figure 2:**
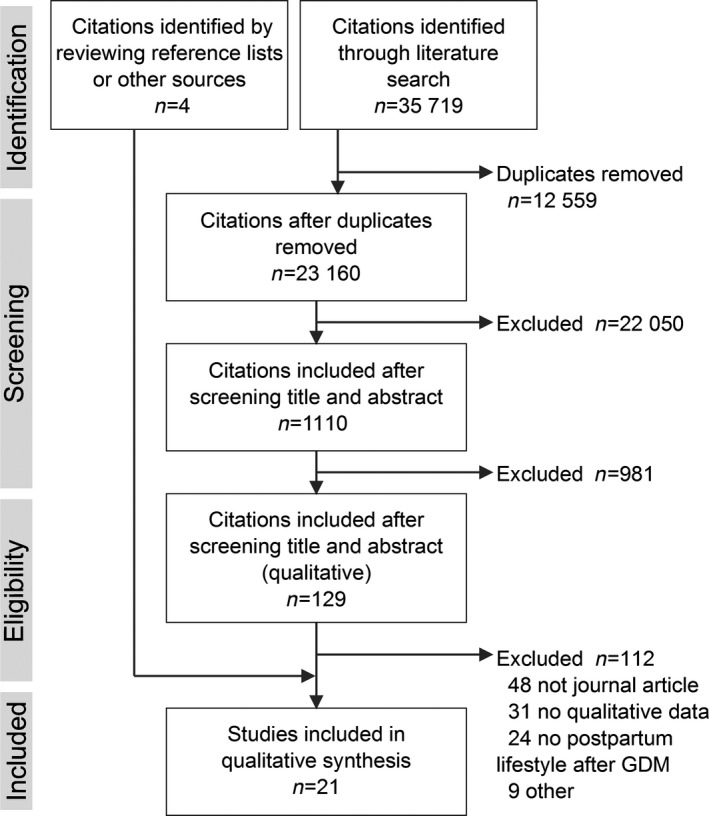
Preferred Reporting Items for Systematic Reviews and Meta‐Analyses (PRISMA) diagram showing number of studies included at each stage of the literature review. GDM, gestational diabetes.

We screened 23 160 citations and reviewed 129 full texts. As shown in Fig. 2 articles were included. Table 1 shows the characteristics of these studies, which together represent the views of 926 postpartum women [median (interquartile range) 17 (11–26) participants per study]. Most included face‐to‐face interviews of women in high‐income countries. Of the 17 studies that specified the timing of data collection, 12 were conducted ≥1 year after the affected pregnancy. The study populations had similar characteristics: women in their mid‐30s who tended to be overweight and have more than one child. Where reported, more than half of the population were employed, married and had higher than secondary education.

**Table 1:** Characteristics of the studies included in the qualitative synthesis

First author and year	Sample size	Setting (country)	Study aim(s) relevant to this analysis	Recruitment strategy	Key inclusion/exclusion criteria	Method of data collection	Time of data collection*	Quality rating (CASP checklist)
Graco 2009 24	10	Australia	To explore perceptions of PA among women with previous GDM, in context of Type 2 diabetes prevention	Purposive sampling (adverts at maternal and child health centres)	hGDM, English‐speaking, age ≥18 years, residence in selected area, not pregnant or since developed Type 2 diabetes	Interviews (not specified)	NR	8.0
Doran 2010 53	11	Tonga	To explore how GDM diagnosis influenced change in diet and PA, influencing factors and support of sustained change	Purposive sampling (hospital records)	hGDM within 1 year, delivered baby at the recruiting hospital	Interviews (face‐to‐face)	Within 1 year	7.0
Evans 2010 37	16	Canada	To determine perceived health status and experiences in establishing and maintaining healthy lifestyle changes	Purposive sampling (GDM clinic)	hGDM, English‐speaking, in the final trimester of pregnancy, telephone access	Interviews (not specified)	At 6 weeks, 3 and 6 months, and 1 year	8.5
Lindmark 2010 36	10	Sweden	To investigate perceptions about lifestyle	Recruited from outpatient endocrinology hospital clinic by mailout	hGDM within 1 year, Swedish‐speaking, age 30–40 years, no other known diseases	Interviews (face‐to‐face)	At 1 year	8.5
Razee 2010 30	57	Australia	To explore beliefs, attitudes, social support, environmental influences etc. on diabetes risk behaviours; preferred forms of programme delivery to inform health promotion	Purposive sampling (GDM hospital clinic databases via letter)	hGDM within 6–36 months, Cantonese‐, Mandarin‐, Arabic‐ or English‐speaking, not pregnant or since developed Type 2 diabetes	Interviews (telephone)	Between 6 months and 3 years	8.0
Bandyopad‐hyay 2011 34	17	Australia	To explore understanding of Type 2 diabetes risk, risk reduction, management strategies, and attitudes and behaviour	Immigrant South Asian women recruited from GDM clinic after diagnosis	hGDM, age ≥18 years, Hindi‐, Bengali‐ or English‐speaking	Interviews (face‐to‐face)	At 6 weeks^†^	8.0
Nicklas 2011 28	25	US	To identify barriers and facilitators to healthy lifestyle changes, and approaches to facilitate participation in interventions	Recruited through flyers and internet postings	hGDM within 7 years, age 18–50 years, English‐speaking, not since developed Type 2 diabetes	Interviews (telephone) and focus groups	Within 7 years	8.5
Gaudreau 2012 40	7	Canada	To understand cultural factors contributing to maintenance of health behaviours encouraged during GDM pregnancy	Recruited by general informants contacts	hGDM within 2–10 years, age ≥18 years, Algonquin peoples, GDM/healthcare in Algonquin community, not breastfeeding or pregnant	Ethnography (observations and interviews)	Between 2 and 10 years	8.5
Hjelm 2012 21	14	Sweden	To explore beliefs about health, illness and healthcare and study their influence on self‐care and care seeking	Consecutive sampling (women born in the Middle East living in Sweden recruited by staff at hospital‐based specialist clinic)	hGDM, age ≥16 years	Interviews (face‐to‐face)	At 3 and 14 months^†^	9.5
Jones 2012 35	17	US	To describe knowledge, perceptions and self‐efficacy beliefs related to preventing cardiometabolic disease	Purposeful and snowball sampling (through fliers distributed by tribal health system care staff)	hGDM, self‐identify as American Indian, age 19–45 years, not pregnant or within 6 weeks postpartum (including 3 with Type 2 diabetes)	Interviews (not specified)	NR	8.0
Dasgupta 2013 22	29	Canada	To identify factors that could enhance participation and engagement in a Type 2 diabetes prevention program	Recruited from GDM clinic via letter from physician (structured recruitment strategy)	hGDM, English‐ or French‐speaking, not pregnant or since developed Type 2 diabetes	Focus groups	Within 5 years	9.0
Lie 2013 32	35	UK	To explore views on postnatal lifestyle change to prevent Type 2 diabetes to inform development of intervention approaches	Purposive then theoretical sampling (diabetes obstetric service contacted by clinic staff while attending appointments or from hospital records)	hGDM within 2 years, English‐speaking, age ≥16 years, successful pregnancy outcome, received antenatal care at specified sites, able to consent	Interviews (face‐to‐face)	Within 2 years then between 12 and 18 months later	8.5
Abraham 2014 33	10	US	To explore lived experiences of women in rural communities with GDM	Purposive and snowball sampling (via obstetric and healthcare providers)	hGDM within 5 years, age ≥18 years, residence in a county eligible for rural community grants, not since developed Type 2 diabetes	Interviews (face‐to‐face and telephone)	Between 2 and 5 years	8.0
Morrison 2014 39	393	Australia	To describe reflections on the experience of GDM‐pregnancy	Australian women recruited from the NDSS database for cross sectional survey by mailout	hGDM within 3 years, age ≥18 years at time of registration, not residing in a Queensland postcode^‡^	Open‐ended survey	At 3 years	7.0
Jones 2015 23	26	USA	To elicit women's perspectives on cardiometabolic risk reduction behaviours to inform the development of a postpartum lifestyle modification intervention	Contact study team after advertising study through fliers and business card distribution at the CNDH	hGDM within 10 years, self‐identify as American Indian, age 19–45 years, healthcare through CNDH	Interviews (face‐to‐face and telephone) and focus groups	Within 10 years (1 or 2 interviews)	8.5
O'Dea 2015 31	17	Ireland	To evaluate a lifestyle intervention programme (give context to quantitative findings)	Women identified from the Atlantic DIP research database and hospital pregnancy service contacted by letters and telephone	hGDM within 1–3 years, English‐speaking, not pregnant or since developed Type 2 diabetes (randomized to the trial intervention arm)	Interviews (face‐to‐face)	Between 1 and 3 years	7.5
Tang 2014 26	23	USA	To explore Type 2 diabetes risk perception and motivators and barriers to preventive health behaviours, to inform intervention approaches	Purposive sampling (African American, Hispanic, non‐Hispanic White women recruited from hospital‐affiliated academic clinics via telephone call from researcher or response to flyer)	hGDM within 1 year, English‐ or Spanish‐speaking, no pre‐existing diabetes or since developed Type 2 diabetes	Interviews (face‐to‐face)	Within 1 year	8.5
Lim 2017 27	165	Australia	To explore the acceptability of a diabetes prevention programme and compare the characteristics associated with programme engagement	Women enrolled in the MAGDA trial	hGDM in most recent pregnancy, English‐speaking, not pregnant, with pre‐existing Type 2 diabetes or other severe illness	Interviews (face‐to‐face and telephone)	NR (1 or 2 interviews)	8.5
Pennington 2017 38	16	Australia	To investigate factors influencing engagement with diabetes preventative care (barriers and enablers), the GP's role in care	Purposive sampling (approached or advertisements at general practices and MCHN centres)	hGDM	Interviews (face‐to‐face and telephone)	NR	8.5
Svensson 2017 25	5	Denmark	To examine the experience of transition from a GDM‐affected pregnancy to postpartum	Random sampling (sent invitation letters via the hospital patient registry and telephoned)	hGDM, recently delivered at the hospital	Interviews (face‐to‐face)	Between 3 and 5 months	8.0
Zulfiqar 2017 29	23	Australia	To explore barriers and facilitators to following long‐term healthy lifestyle recommendations, and whether there were differences between overseas‐born‐ and Australian‐born‐women	Women managed by a hospital DIP Service who attended a GDM‐related health education programme	hGDM, English‐speaking, live singleton delivery, not pregnant or since developed Type 2 diabetes	Interviews (face‐to‐face)	More than 3 years	8.5

CASP, Critical Appraisal Skills Programme (score out of 10); CNDH, Chickasaw Nation Department of Health; DIP, Diabetes in Pregnancy; GDM, gestational diabetes; GP, general practitioner; hGDM, history of gestational diabetes; MAGDA, Mothers After Gestational Diabetes in Australia, MHCN, maternal and child health nurse centres; NDSS, National Diabetes Service Scheme; NR, not reported; PA, physical activity.

^*^ reference to/since gestational diabetes‐affected pregnancy (studies collected data once postpartum unless otherwise specified); ^†^Plus 1 during pregnancy; ^‡^Due to a concurrent study.

If reported, healthier diets usually involved trying to consume more fruit and vegetables, and less sugar, fat and processed foods by making substitutions: for example, ‘…I take light milk… We have changed… so it's low‐fat…’ 21. Walking was most frequently mentioned because it ‘…is the easiest exercise you can do’ 22, and several participants mentioned running.

We found all of the studies to be good quality (mean CASP score 8.0/10; Table S2). All were appropriate for qualitative methods, with clear aims, results and implications. Generally, data collection was suitable, although sometimes important details were missing: authors rarely commented on their relationship with participants or their implementation of ethical procedures, even though approval had been granted. Mixed methods studies scored lower because qualitative aspects were less well reported or fitted around quantitative methods.

Actual and anticipated barriers to and facilitators of healthy postpartum lifestyle were translated into six themes that are described below and summarized in Table 2, alongside a seventh theme covering views on practical aspects of interventions. The studies contributing to each theme are shown in Table S3. We did not include a theme regarding culture but discussed it in context.

**Table 2:** Summary of themes developed in the qualitative synthesis

Theme	Description	Consequences for healthy lifestyle	Illustrative quotations
Role as mother and priorities	Women's *identity* was as a mother, requiring them to *prioritize* their family; most *guilt* was felt for not doing this	This was a barrier when giving families what they wanted and not having time for themselves, or a facilitator when health was recognized as important for their family	‘[My child] already goes to occasional care on Friday mornings… but that's mainly so I can do the housework… the thought of putting him in care so I can do exercise, yeah, that's a big guilt on me’ 24 ‘I don't [change my eating habits] so much for protecting me from getting diabetes; I do it so that my son, as he is learning to eat, he learns to eat healthier’ 26
Support from family and friends	*Family* could provide support by reducing burdens and, particularly affecting diet, providing information and being involved. *Friends* could offer encouragement for exercise and make it more pleasant. *Societal/cultural norms* influenced ability to have a healthy diet	Having support facilitated healthfulness; absence of support was identified as barrier	‘Maybe [you need] help from your significant other because it's hard when they are eating cake and ice cream, all the stuff you can't have, and maybe just don't even have it in the house’ 33 ‘If the other women can do it so can I. If others with three children can exercise, I with one can also change’ 27
Demands of life	Lack of *time* and *energy*,* busyness* and *work* influenced lifestyle choices, as did how *convenient* and easy to *integrate* into daily life it was	This was mainly a barrier to healthy lifestyle, although sometimes healthy options became part of daily life and saved time	‘I was exhausted and already feeling so guilty for being away from my child while I was working, so I did not exercise’ 28 Meal planning ‘to reduce the number of trips per week to grocery stores’ 22
Personal preferences and experiences	Food played an important *role* in women's personal and social lives. Both diet and exercise affected *emotions*	Behaviour was determined by whether women had positive experiences or benefitted from healthy/unhealthy lifestyles	‘Everything's back to normal so I've sort of been making up for lost time a little bit with all the chocolate I couldn't have’ 32 ‘…If I'm not active then I find I don't cope as well with things’ 24
Diabetes risk perception and information	Women learned about diet during their GDM‐affected pregnancy; knowledge included *risk of Type 2 diabetes*, how to prevent it, repetition of messages and the need for *culturally relevant* information	Relevant information facilitated healthfulness; absence of information was identified as a barrier	‘The women felt neglected by healthcare providers and were left with unanswered questions about what to do next’ 37 ‘…So the plan is to try and live healthy, get rid of the extra pregnancy kilos and return to my normal weight again, and then to be physically active’ 25
Finances and resources	*Resources* were needed to help women sustain a healthy lifestyle, and their lifestyle affected the family's *finances*	Women thought that more resources would help them to be more healthy	‘…[Healthy foods] are not the cheap items; they're a kind of more in the pricy end. It could be a bit irritating to prioritise your money in that way…’ 25 ‘I didn't eat out as often. It became less expensive to eat out because I cut down on my portions’ 40

Italic highlights key components of the themes (subthemes). GDM, gestational diabetes.

### Role as mother and priorities

Prioritizing children and trying to be what the women perceived to be a good mother had one of the greatest influences on their views of healthy postpartum behaviour; preventing diabetes was rarely the primary motivation.

Many women's principle identity was as a mother and partner (‘matriarch’ 23), which meant responsibility for childcare, housework and food, and they wanted to do a ‘good job’. Specifically, many found it difficult to exercise while with a child because they needed or wanted to care for them: ‘[My child] already goes to occasional care on Friday mornings… but that's mainly so I can do the housework… the thought of putting him in care so I can do exercise, yeah, that's a big guilt on me’ 24. Healthy lifestyle could become less important after pregnancy because it was ‘no longer seen as having a direct impact on the child’ 25. Conversely, others thought they should role model healthy behaviour, provide healthy food and maintain their own health in order to care for their children: ‘I don't [change my eating habits] so much for protecting me from getting diabetes; I do it so that my son, as he is learning to eat, he learns to eat healthier’ 26.

Similarly, mothers often prioritized their family's preferences or finances. Some experienced objection when they cooked healthy foods or thought that not eating their traditional diet jeopardized family identity (‘…chang[ed] the culture of the food…’ 22), although in some cases the whole family's diet changed to prioritize children's health. Some even thought that it was ‘inappropriate’ to consider exercise while caring for a small child: ‘All my time is devoted to them now…’ 24. Conversely, some participants in the study by Lim *et al*. 27 planned how to overcome challenges and prioritized attendance at a diabetes prevention programme: ‘I gave up working on Thursdays to come’. For these reasons, many wanted to include their families and children in healthier lifestyles or programmes.

Resulting from this strong identity, guilt was common across several themes. Some felt ‘a moral tug’ 23 if they left children or housework in order to exercise or attend a programme, and did not see these as legitimate reasons to use external childcare. They also felt guilty for inconveniencing their wider family when they believed they should do childcare, even if help was offered. In contrast, others felt guilty for not exercising when they thought they should.

### Support from family and friends

Support was an important facilitator to healthy behaviour whereas its absence was a barrier, considering the support‐giver's own knowledge and diabetes risk perception. Family could help with childcare or housework to reduce busyness and tiredness, and encourage exercise: ‘[The partner needs to consider that] if I don't help with this then [the mother] might be too tired to actually get out for the run she actually would like to go for…’ 25. In particular, family could be a source of information about healthy diet such as the nutritional content of food. They needed to support and join in eating healthily: ‘They'll tease you about how you can't eat this food, and they put it in front of you… try to get you to eat it’ 23 and ‘[I would need] family on board because I can't make two separate meals’ 28. Additionally, more support was expected if partners attended part of the intervention: ‘…I can explain to him really what's going on but if he would hear it from elsewhere, maybe, it'll be different’ 22.

Peer support encouraged exercise, which became an opportunity for socializing: ‘I like having a buddy system. I've never liked to do exercise on my own…’ 22. General lack of support, particularly in migrant populations, could result in isolation, depression and abandonment because women avoided social eating or dropped their diets in certain situations 29. Arabic‐speaking women ‘felt duty bound to eat whatever was offered to them when they visited their family or friends. Such cultural expectations “created more problems” even when the family or friends’ intention was to be helpful’ 30.

Women valued social support from programmes. They motivated and shared experiences with fellow participants ‘…because we're all in that group together’ 23, and clinicians or programme facilitators provided further accountability. Some continued this supportive relationship beyond the programme. O'Dea *et al*. 31 reported that childcare was the biggest barrier to attending lifestyle interventions, women without a partner could not attend, and the partner needed to support attendance.

### Demands of life

Affected by the maternal role and limited support, lack of time and energy were key barriers to healthy behaviour. Specifically, these were barriers to thinking about, preparing for and doing exercise, and planning and cooking healthy meals: ‘You're so busy and so tired and the last thing you want to be bothered thinking about is whether you're eating properly and exercising enough’ 32 and changes could go ‘by the wayside’ when ‘you get busy’ 33. In particular, physical activity was frequently viewed as distinct from the other parenting demands: for many it required ‘set[ting] aside time’ 34 and ‘taking time out for themselves’ 24 away from children and housework (their priorities). They needed to preserve energy, not use it on exercise. Alternatively, physical activity became more sustainable when it became a ‘daily habit’ 31, such as a mother ‘always walk[ing] upstairs to change her baby's diaper’ 28. Similar views were held when considering attending a programme, particularly if the woman needed to travel or the time was inconvenient.

Although shopping with children was difficult, healthy diet did not have as big an impact on time because the role of a mother already included cooking. Furthermore, some reported saving time through meal planning, such as ‘to reduce the number of trips per week to grocery stores’ 22.

Similarly, work increased busyness and created opportunities for unhealthy eating, such as canteens and because ‘[sweets] are often available at work. Meetings have danishes and muffins, cheese plate’ 28. Work also took women away from their children, exaggerating the feelings of guilt and the desire not to access childcare in order to exercise.

Finally, a healthier lifestyle was thought to be hard because of the possibility of saving time and inconvenience through unhealthy options. Using the car was easier than walking, and unhealthy ready meals were easily available.

### Personal preferences and experiences

Behaviours were also influenced by personal perspectives and previous experiences. Food was considered as an important part of life. Acting as a barrier to healthy eating, food was a key aspect of many get‐togethers and celebrations: the ‘…highlight of any kind of social gathering is that you've got to have food to celebrate’ 35. Furthermore, some women viewed unhealthy food as a pleasure, reward or comfort. For example, home cooking made a South Asian woman living in Australia ‘…feel closer to your home and that you still have this power and that you're still free to choose…’ 29. Some considered it their right to eat what they wanted. Breastfeeding led to additional hunger and eating more; some craved food such as chocolate. Conversely, other participants enjoyed feeling healthier on certain diets.

Some women reported positive experiences that helped them to maintain exercise: it relaxed and energized them, reduced stress, and helped them to eat a healthy diet. Conversely, others did not enjoy exercise (‘I find it so boring’ 36) or struggled to exercise in bad weather.

### Diabetes risk perception and information

Perception of diabetes risk varied. Women in most studies were aware of the link between GDM and Type 2 diabetes but many did not recognize their personal risk 21, 25, 26, 28, 29, 32, 33, 36, 37, 38, thinking that they could go ‘back to normal’ 29 rather than make lifestyle changes. Some demonstrated a lack of understanding (‘I am confident. Nobody in my family ever had it… [explains her lifestyle]’ 26) or had misleading information that the diabetes risk was in the past.

Conversely, others were worried about developing diabetes, which they viewed as inevitable (‘…there's not a great lot more I can do’ 32) or motivated attempts to delay (rather than prevent) it through lifestyle changes, particularly if women were familiar with diabetes through affected friends or family. Nevertheless, strong risk perception influenced willingness rather than ability to make changes. In this case, the risk tended to be considered in the future (‘I feel like I still have time to make changes down the track’ 39); others were continuously aware (‘The risk of getting diabetes is in the back of your mind, you think about what to eat and to exercise, struggling to reduce weight. It is really that simple but also so hard’ 36), even if risk perception declined over time as life ‘moved on’ 37.

Lack of information was reported in most studies. After the intense monitoring of pregnancy, women felt ‘abandoned’ 25, 37, 39, ‘…left high and dry’ 32, and were ‘neglected by healthcare providers and were left with unanswered questions about what to do next’ 37. Some could not remember the health messages after delivery, while a few of those that heard the same information again found it either annoying or said ‘…even if it is old knowledge it is good to hear it once more’ 36. Some women focused on diet to lose weight to prevent diabetes, and used dietary advice from their GDM‐affected pregnancy postpartum. Diabetes prevention programmes were considered useful for learning about diabetes, exercise, diet and weight loss.

Women appreciated information that was relevant to them, particularly information that was culturally appropriate. Algonquin women benefitted from help to adapt their traditional diet by switching cooking oil or using alternative meats. Furthermore, the information was delivered appropriately because ‘…they intervened immediately, adapting to a culture‐specific concept of time described by the general informants as “now or never” 40. Irrelevant information was not useful; for instance, women then became torn between healthier diets and maintaining cultural identity.

### Finances and resources

Lack of resources and the need to prioritize finances were frequently cited as barriers to healthy behaviour. Healthy lifestyles were perceived as more expensive than unhealthy ones: healthy food was more expensive than junk food and going to the gym was more expensive than not exercising (particularly when external childcare was needed). Gyms, if available, were also seen to take up time and keep them away from children; none reported being able to use them. Access to cheaper or free healthy food and facilities, and resources such as recipes and home exercise equipment or DVDs were expected to increase healthiness. Gaudreau and Michaud 40 found that women could sustain a healthier diet because they found that it was cheaper: ‘I didn't eat out as often. It became less expensive to eat out because I cut down on my portions’.

### Format of interventions

Finally, ‘social support and promot[ing] family participation’ 23 were more important than how diabetes prevention programmes or interventions were delivered. Web‐based interventions were flexible, which could help with time and childcare barriers and allow provision of support and encouragement; however, some wanted face‐to‐face contact or not to spend more time on computers. Telephone call interventions were not popular, despite women in the study by Lim *et al*. 27 finding that they were personal and flexible, but one population preferred text messages 23. The greatest appeal of face‐to‐face interventions was that they could provide social support, including accountability, motivation and fulfilling social needs. However, mental health could be a barrier to group settings: one woman found it awkward to discuss and another did not attend because she had depression 27. Mixed interventions were suggested to obtain the benefits of multiple approaches: ‘a peer group in‐person to start, to get to know each other, then use chat rooms/email to access at all times of the night’ 28.

Little was discussed about the preferred timing for intervention. Participants in the studies by Dasgupta *et al*. 22 and Jones *et al*. 2015 23 reported that the intervention should start during pregnancy or immediately postpartum to address feeling unsupported after pregnancy. Conversely, Lie *et al*. 32 concluded that the weaning period provided a ‘window of opportunity’.

Several considered that lifestyle coaches, trainers or counsellors could provide support, while medical staff were seen as a trustworthy source of knowledge, but the studies did not discuss who should deliver a programme.

### Recommendations for promoting behaviour change

In light of our findings, we developed 20 recommendations for promoting healthier lifestyles after GDM (Table 3) and mapped these onto the behaviour change technique taxonomy to suggest a range of behaviour change techniques that could be included in future interventions, if appropriate to the setting. To illustrate, recommendation 7 (‘provide guidance about how to buy and prepare healthy, tasty food efficiently’) is a ‘10.6 Non‐specific incentive’ in itself that incentivizes women to save time and money through dietary changes. The physical activities suggested in recommendation 17 could be implemented through ‘1.1 Goal setting (behaviour)’ by helping women to create personal daily walking targets or playing with their children at the park four times a week rather than sitting and watching.

**Table 3:** Twenty recommendations for promoting healthier lifestyles after gestational diabetes, and our confidence in each recommendation made using the GRADE‐CERQual approach

Recommendation	Behaviour change techniques relevant to recommendation 19	Confidence in evidence and explanation
**Role as mother and priorities**		
Highlight the benefits to the family of the mother being healthier and role modelling healthy lifestyle to children as the incentive for change, alongside preventing diabetes	5.1 Information about health consequences, 5.3 Information about social and environmental consequences, 10.5 Social incentive, 10.7 Self‐incentive, 13.1 Identification of self as role model	**Moderate:** Women directly or indirectly reported that their children were their incentive for change; whether it is appropriate for all should be considered
Include the option of childcare in face‐to‐face interventions if children are not part of the sessions	12.2 Restructuring the social environment, 14.1 Behaviour cost	**Moderate:** Few studies contributed to this recommendation but some directly suggested it; it is supported by general concern about children/childcare
**Support from family and friends**		
Promote healthier lifestyles in the wider family (and friends)	7.3 Reduce prompts/cues, 12.2 Restructuring the social environment	**Moderate:** It is clear that women need support for a healthy diet but few studies clearly discussed family and friends exercising
Encourage the wider family (and friends) to promote healthy lifestyles in mothers and support them practically (such as relieving housework burdens)	3.2 Social support (practical), 3.3 Social support (emotional)	**High:** Many studies explained the benefits of or need for support for lifestyle change
Include the family in interventions (e.g. information or modules for partners and children)	3.2 Social support (practical), 3.3 Social support (emotional)	**Moderate:** Inadequate data reduced our confidence that this recommendation would be useful to postpartum women
Encourage and facilitate women to exercise with others/a buddy	3.3 Social support (emotional)	**Moderate:** This recommendation was developed from the general need for support, plus a few studies that specifically addressed it
**Demands of life**		
Provide guidance about how to buy and prepare healthy, tasty food efficiently	1.2 Problem solving, 4.1 Instruction on how to perform a behaviour, 10.6 Non‐specific incentive	**High:** Many women reported the lack of and need for more guidance for having a healthy diet
Provide guidance about how to exercise around the house and as part of regular daily routines	4.1 Instruction on how to perform a behaviour, 8.3 Habit formation, 10.6 Non‐specific incentive	**Moderate:** It is clear, and stated, that women need help to increase exercise; however, there are some contradictory suggestions about the best form(s) of exercise to promote and how
**Personal preferences and experiences**		
Support women to maintain healthy behaviour/diet in challenging situations, e.g. social gatherings, breastfeeding, at work (particularly for vulnerable groups)	1.2 Problem solving, 1.4 Action planning, 4.2 Information about antecedents	**Low:** Certain situations affect women's ability to maintain healthy diets; the best way to address this is unclear
Highlight the wider benefits of healthier lifestyle (such as reducing stress and weight as well as diabetes risk)	9.2 Pros and cons, 9.3 Comparative imagining of future outcomes, 13.2 Framing/reframing	**High:** Women had identified many benefits of adopting healthier lifestyles that helped them to maintain them (perhaps after awareness of diabetes risk declined over time)
**Diabetes risk perception and information**		
Make information, resources and training easily accessible and make interventions available to start immediately after pregnancy (or during pregnancy)	4.1 Instruction on how to perform a behaviour, 5.1 Information about health consequences, 5.2 Salience of consequences	**High:** This recommendation resulted from many studies that were in agreement, with few exceptions
Ensure that interventions are culturally appropriate and recommendations allow maintenance of women's identity	13.2 Framing/reframing, 13.5 Identity associated with changed behaviour	**High:** It was clear that women wanted culturally relevant interventions and that they were beneficial to those who received it
Ensure that care providers consider women's attitude towards diabetes and advise them on their risk appropriately	5.1 Information about health consequences, 5.2 Salience of consequences	**Low:** This recommendation is a step on from women's attitudes towards behaviour change and their clinician
Promote a long‐term perspective about maintaining healthy lifestyle, with an ‘every little helps’ approach, rather than ‘all or nothing’, and include the importance of both diet and activity	5.1 Information about health consequences	**Moderate:** Paucity of data reduced our confidence in this recommendation
**Finances and resources**		
Provide information about low‐cost or money‐saving healthy behaviours and resources; interventions should be free	4.1 Instruction on how to perform the behaviour	**High:** There was agreement across studies but this was not reported in detail
**Format of intervention and other**		
Recommend increasing fruit and vegetable intake, reducing sugar and substituting with healthier ingredients or methods to improve diet	1.1 Goal‐setting (behaviour), 1.4 Action planning	**Moderate:** Several studies briefly reported women being able to makes these changes
Recommend flexible exercise such as walking and those performed around the home or with the baby to increase physical activity (rather than attending gyms or classes)	1.1 Goal‐setting (behaviour), 1.4 Action planning	**High:** Women across several studies reported how and why they did these types of exercises
Ensure interventions have web‐based components but encourage additional face‐to‐face contact (they should not depend on women attending sessions)	6.2 Social comparison	**Low:** There was no agreement across studies; this recommendation attempted to consider what women wanted but also what was most practical
Deliver and promote interventions from recognized/trusted sources (eg. the healthcare provider or a dietitian)	9.1 Credible source	**Low:** Preferred source of the intervention was not discussed; however women reported benefits from their interactions with various professionals
Promote establishment of systems to monitor progress and accountability (through an intervention or ensure the participant establishes this themselves)	2.2 Feedback on behaviour, 2.3 Self‐monitoring of behaviour, 2.4 Self‐monitoring of outcome of behaviour, 3.2 Social support (practical)	**High:** Accountability facilitates behaviour change, but the best way to promote this remains uncertain

Recommendations frequently result from findings within multiple themes but have been presented under the primary contributing theme.

As explained in Table S4, we had high confidence in 8 recommendations, moderate confidence in another 8 recommendations and low confidence in 4 recommendations in the GRADE‐CERQual evaluation. The recommendations were based on many good‐quality, relevant studies; confidence was therefore largely influenced by coherence and agreement between studies and richness of the data. We tended to have greater confidence about information that women wanted and the need for support and accountability, but less confidence in recommendations about equipping women in situations such as at work, the behaviour of friends and family (other than offering support) and interactions with professionals because continued contact is not common. We felt that it was important to adapt interventions to the target population and facilitate family‐friendly changes because the mother's own diabetes risk was unlikely to motivate change without her perceiving benefits for her children. Some of the most beneficial aspects of groups (such as forming supportive relationships) mean that they are impractical for most to commit to in the long term. Consequently, a combination of approaches could be most appropriate; for example, online information, target‐setting and accountability, plus options to arrange video calls with dietitians and connections with local mothers’ groups.

## Discussion

Adopting a healthy lifestyle after a pregnancy affected by GDM is complex. Their identity as a mother who prioritized family above themselves influenced many women's ability to care for their own health, as did the need for resources, time, energy, information and support. Taking into consideration the significant impact that having new children has, these barriers frequently appeared to outweigh the perceived benefits of behaviour change by those maintaining established unhealthy behaviours, particularly when a negative effect on the family was anticipated.

Influences on behaviours were similar, although a diet could be adapted because meal preparation and eating were already necessary, whereas exercise was an additional task. Some influences were both positively and negatively reported; for example, lack of culturally specific information inhibited healthy diet (information as a barrier), while guidance about adapting traditional foods helped women to make changes (information as a facilitator). In contrast, some facilitators were only anticipated; for example, women suggested giving gym passes to increase exercise, but none reported regularly using gyms.

### Strengths and limitations

Strengths of the present study include the fact that it is the first comprehensive qualitative synthesis to focus on the views of women with a history of GDM on having a healthy lifestyle, and to make clear recommendations for implementing the findings. As a multidisciplinary team, we conducted a comprehensive literature search and thematic synthesis to identify repeated themes across studies and to recognize those that may have previously been overlooked 18. Our concurrent comparison of positive and negative influences and different behaviours permitted a more representative understanding. We observed diverse perspectives and variety between and within study populations (such as ethnicity, social norms, other children and family members). Congruence between high‐quality studies increased our confidence in our recommendations, which were transparently evaluated using GRADE‐CERQual and linked to standard behaviour change techniques.

The study also has some limitations. We did not distinguish between time points but collated studies that collected data from 6 weeks to 10 years postpartum so we could not synthesize changes over time as reported by Hjelm *et al*. 21. Furthermore, we were not able to investigate specifically how experience of pregnancy, such as struggling to manage diabetes through lifestyle modifications or feeling guilty for having GDM 5, influenced postpartum behaviour based on the included studies. Most data were from educated or employed women recruited from medical settings in developed countries, meaning that we missed some experiences of motherhood (although the populations were quite different, as discussed). Although it is possible that participants felt that mental health did not influence behaviour, it is also possible that they avoided this topic and that women experiencing mental health difficulties did not participate in these studies. We did not access the primary data therefore were reliant on how the studies’ authors interpreted and reported their data, nor did we examine quantitative literature. Barriers made the greatest contribution to analytical themes, perhaps because they were emphasized by researchers or respondents. Fewer studies reported experiences of diabetes prevention programmes, but they were consistent with other themes.

Although the studies were good quality, quality did affect the results of the synthesis and recommendations. Authors rarely adequately considered their role as researchers, which could have led to bias in the formation and evaluation of research questions and social desirability bias among respondents. Furthermore, although we did not influence the participants or original analyses, our analysis was inevitably affected by our own preconceptions. In recognition of this, we developed the coding frame from the study findings in order not to impose a framework from our review question, used structured CASP and GRADE‐CERQual checklists, and all authors discussed the themes and findings.

### Comparison to other studies

Whilst our findings are broadly consistent with previous literature reviews, we have added more studies, data and detail. In 2014, a meta‐synthesis found that, in the context of preventing diabetes in the future, women prioritized children and families and listed barriers and facilitators 6. The authors of that paper noted that few studies contributed to this, whereas we identified 11 more studies published since their search. Two other reviews, which had a greater focus on healthcare seeking, commented that many women have knowledge regarding diabetes prevention that affects their desire to live healthily 14, 16. They also list numerous barriers, including some that we found less emphasis on, such as poor body image and an unsuitable neighbourhood. Consistent with our findings, a discussion of a recent symposium concluded that postpartum behaviour is affected by women's beliefs about their susceptibility to diabetes, and is considered at the cost of their family, and that healthcare systems gave disjointed care so women lacked information 41.

Postpartum mothers in the general population also report barriers to physical activity including lack of energy, time for housework and the responsibility of childcare 42, 43. In the study by Graco *et al*. 24, women with GDM did not want to be seen as a separate group but to attend classes with mothers who had had a normoglycaemic pregnancy. This raises the question of whether interventions should be specifically targeted at women with previous GDM or mothers seeking healthy lifestyles in general. Our results also broadly agree with the determinants of healthy behaviour in the wider adult population, although we think that there is a different emphasis: mothers with previous GDM appear to weigh relational factors (such as the possible impact of their behaviour on others) higher than other populations and place less emphasis on environmental factors and personal health benefits 44.

Moreover, our recommendations are similar to those identified in the development of the STAR MAMA intervention 45. In that study, focus groups (including overweight women or those with GDM), alongside experts, were used to adapt the DPP to Latina women through the behaviour change wheel framework. In the adapted programme, techniques such as modelling narratives and role‐playing were used to help participants with a history of GDM overcome barriers to behaviour change through automated weekly telephone calls and coaching. The initial evaluation of the intervention was positive 46.

### Implications

As outlined in Table 3, the present qualitative review can inform approaches to promoting healthier lifestyles. These recommendations could be used to develop new interventions or adapt existing ones. For example, the effective DPP intensive lifestyle intervention focused on repeated face‐to‐face meetings with a case manager 13; given our findings, this could make it hard for many women to commit to (the DPP has already been adapted for the STAR MAMA intervention 46). Total diet replacement and stepped food reintroduction in a population with diabetes (DiRECT trial) resulted in diabetes remission in half of their participants 47, but a diet that is so controlled and different from that of the rest of the family may not be attractive to mothers. Web‐based interventions with additional face‐to‐face or remote support from a nurse (POWeR+ trial) have led to weight loss in the general population 48, and could be adapted to meet the specific requirements of this population.

We have also identified areas that need further research. Despite including a number of recent studies, we were not able to examine the use of technologies such as smartphone applications and social media, which are growing across the world. In a study that was published after we conducted our literature search, participants suggested that more support should be provided via online forums and information on general practice websites 49. The authors of that study reported that technology could provide information, enable personalized self‐management and meet social needs, with flexibility noted as a benefit. Additionally, we were unsure whether promoting change in the wider family would specifically facilitate mothers to be healthier based on this review; however, the risk of diabetes is higher in partners and children of mothers with GDM 50, 51 and maternal behaviour strongly correlates with childhood obesity 52, therefore, it should be carefully considered.

Furthermore, careful attention should be given to how best to apply these recommendations. For example, interventions could be tailored to working and single mothers or those experiencing postpartum mental health disorders, and the appropriateness of using additional behaviour change techniques (such as ‘14. Scheduled consequences’ 19).

### Conclusion

In conclusion, many factors make it difficult to adopt healthy lifestyles after GDM, yet how women interpret these factors can motivate or prevent changes that reduce their diabetes risk. Women's needs and experiences should be considered when designing strategies to promote healthier lifestyles. We have made key recommendations based on a synthesis of qualitative data that will inform the development of feasible interventions, or adaption of existing ones, to educate and support women in achieving and maintaining a healthy postpartum lifestyle in order to reduce their risk of developing Type 2 diabetes.

### Funding sources

R.A.D. is funded by a PhD studentship from the National Institute for Health Research (NIHR) School for Primary Care Research (SPCR). This paper presents independent research funded by the NIHR SPCR. The views expressed are those of the author(s) and not necessarily those of the NIHR, the NHS or the Department of Health. R.J.W. is funded by an NIHR Academic Clinical Fellowship. S.J.G. is supported by the Medical Research Council (MC_UU_12015/4). The University of Cambridge has received salary support in respect of S.J.G. from the NHS in the East of England through the Clinical Academic Reserve. J.U‐S. is funded by a Cancer Research UK Cancer Prevention Fellowship (C55650/A21464).

### Competing interests

None declared.

## Supporting information


**Table S1.** Medline search strategy.
**Table S2.** Findings from the Critical Skills Appraisal Programme (CASP) checklist.
**Table S3.** Studies contributing to each theme.
**Table S4.** CERQual qualitative evidence profile of recommendations for promoting healthy lifestyles after gestational diabetes.
