# Supplementary material for: Women's views on lifestyle changes to reduce the risk of developing Type 2 diabetes after gestational diabetes: a systematic review, qualitative synthesis and recommendations for practice
Source: Diabet Med. 2019 Mar 4;36(6):702–17. doi: 10.1111/dme.13926 (PMC6563496; doi:10.1111/dme.13926)
Supplement: Supplementary file 1 — Table S1. Medline search strategy. Table S2. Findings from the Critical Skills Appraisal Programme (CASP) checklist. Table S3. Studies contributing to each theme. Table S4. CERQual qualitative evidence profile of recommendations for promoting healthy lifestyles after gestational diabetes. [file DME-36-702-s001.pdf]

Supplementary material

SUPPLEMENTARY TABLE 1: Medline search strategy

1.	type 2 diabetes.mp. or Diabetes Mellitus, Type 2/
2.	T2DM.mp.
3.	NIDDM.mp. or Diabetes Mellitus, Type 2/
4.	non insulin dependent diabetes.mp.
5.	glucose tolerance.mp.
6.	insulin resistance.mp. or Insulin Resistance/
7.	1 or 2 or 3 or 4 or 5 or 6
8.	gestational diabet*.mp.
9.	diabetes in pregnancy.mp.
10.	Pregnancy/ or pregnancy.mp.
11.	type 2 diabet*.mp.
12.	10 and 11
13.	gestation*.mp.
14.	11 and 13
15.	postpartum diabet*.mp.
16.	postpartum.mp. or Postpartum Period/
17.	8 or 9 or 12 or 14 or 15 or 16
18.	prevent*.mp.
19.	progress*.mp.
20.	develop*.mp.
21.	advanc*.mp.
22.	incidence.mp. or Incidence/
23.	avoidance.mp.
24.	prohibit.mp.
25.	establish.mp.
26.	health promotion.mp. or Health Promotion/
27.	Exercise/ or exercise.mp.
28.	active living.mp.
29.	metformin.mp. or Metformin/
30.	weight.mp. or "Weights and Measures"/
31.	risk factors.mp. or Risk Factors/
32.	Insulin/ or insulin.mp.
33.	exercise therapy.mp. or Exercise Therapy/
34.	intervention.mp.
35.	interven*.mp.
36.	yoga.mp. or Yoga/
37.	postnatal.mp.
38.	diet.mp. or Diet/
39.	healthy eating.mp. or Healthy Diet/
40.	behaviour.mp.
41.	physical activity.mp. or Exercise/
42.	lifestyle.mp. or Life Style/
43.	manag*.mp.
44.	screening.mp. or Mass Screening/
45.	hypoglycaemic agents.mp.
46.	hypoglycaemics.mp.
47.	health promotion.mp. or Health Promotion/
48.	medication.mp.
49.	medical therapy.mp.
50.	rate.mp.
51.	predictor*.mp.
52.	risk*.mp.
53.	factor*.mp.

54.	18 or 19 or 20 or 21 or 22 or 23 or 24 or 25 or 26 or 27 or 28 or 29 or 30 or 31 or 32 or 33 or 34 or 35 or 36 or 37 or 38 or 39 or 40 or 41 or 42 or 43 or 44 or 45 or 46 or 47 or 48 or 49 or 50 or 51 or 52 or 53
55.	follow-up.mp.
56.	postpartum.mp. or Postpartum Period/
57.	qualitative.mp.
58.	Interview/ or interview.mp.
59.	focus group*.mp.
60.	health service.mp. or Health Services/
61.	belief*.mp.
62.	opinion*.mp.
63.	survey.mp.
64.	54 or 55 or 56 or 57 or 58 or 59 or 60 or 61 or 62 or 63
65.	7 and 17 and 64

SUPPLEMENTARY TABLE 2: Findings from the Critical Skills Appraisal Programme (CASP) checklist

Study		1. Clear statement of aims?	2. Qualitative methodology?	3. Appropriate research design?	4. Appropriate recruitment strategy?	5. Suitable data collection?	6. Researcher-participant relationship considered?	7. Ethical issues considered?	8. Rigorous data analysis?	9. Clear findings?	10. Valuable to us?	Score (/10)
**Graco 2009**		🟢	🟢	🟢	🟢	🟢	🔴	🟡	🟡	🟢	🟢	8.0
**Doran 2010**		🟢	🟢	🟢	🟢	🟢	🔴	🟡	🔴	🟡	🔴	6.0
**Evans 2010**		🟢	🟢	🟢	🟢	🟢	🔴	🟡	🟢	🟢	🟡	8.0
**Lindmark 2010**		🟢	🟢	🟢	🟡	🟢	🔴	🟢	🟢	🟢	🟡	8.0
**Razee 2010**		🟢	🟢	🟢	🟢	🟢	🔴	🟡	🟢	🟡	🟢	8.0
**Bandyopadhyay 2011**		🟢	🟢	🟢	🟢	🟢	🔴	🟡	🟡	🟢	🔴	7.0
**Nicklas 2011**		🟢	🟢	🟢	🟢	🟢	🟡	🟡	🟡	🟢	🟢	8.5
**Gaudreau 2012**		🟢	🟢	🟢	🟡	🟢	🟡	🟡	🟢	🟢	🟢	8.5
**Hjelm 2012**		🟢	🟢	🟢	🟢	🟢	🟡	🟢	🟢	🟢	🟡	9.0
**Jones 2012**		🟢	🟢	🟢	🟢	🟢	🔴	🟡	🟢	🟡	🟡	7.5
**Dasgupta 2013**		🟢	🟢	🟢	🟢	🟢	🟡	🟡	🟢	🟢	🟢	9.0
**Lie 2013**		🟢	🟢	🟢	🟢	🟢	🔴	🟡	🟢	🟢	🟢	8.5
**Abraham 2014**		🟢	🟢	🟢	🟡	🟢	🔴	🟡	🟢	🟢	🟢	8.0
**Morrison 2014**		🟢	🟡	🟡	🟢	🟡	🔴	🟢	🟢	🟡	🟡	6.5
**Jones 2015**		🟢	🟢	🟢	🟢	🟢	🔴	🟡	🟢	🟢	🟢	8.5
**O'Dea 2015**		🟢	🟢	🟢	🟡	🟢	🔴	🟡	🟡	🟢	🟢	7.5
**Tang 2015**		🟢	🟢	🟢	🟢	🟢	🔴	🟡	🟢	🟢	🟢	8.5
**Lim 2017**		🟢	🟢	🟢	🟢	🟢	🟢	🟡	🟢	🟡	🟡	8.0
**Pennington 2017**		🟢	🟢	🟢	🟢	🟢	🔴	🟡	🟢	🟢	🔴	7.5
**Svensson 2017**		🟢	🟢	🟢	🟡	🟢	🔴	🟢	🟢	🟢	🟡	8.0
**Zulfiqar 2017**		🟢	🟢	🟢	🟢	🟢	🟡	🟡	🟡	🟢	🟢	8.5
Score frequency	Yes	21	20	20	16	20	1	4	15	16	11	
	Unclear	0	1	1	5	1	5	17	5	5	7	
	No	0	0	0	0	0	15	0	1	0	3	

Green dot: yes (1 point); yellow dot: can't tell/unclear (0.5 points); red dot: no (0 points)

<u>SUPPLEMENTARY TABLE 3</u>: Studies contributing to each theme

Study	CASP score	Role as mother and priorities	Support from family and friends	Demands of life	Personal preferences and experiences	Diabetes risk perception and information	Finances and resources	Format of interventions
**Graco 2009**	8.0	●		●	●	●		●
**Doran 2010**	6.0					○		○
**Evans 2010**	8.0	○		○		●	○	
**Lindmark 2010**	8.0				●	●		
**Razee 2010**	8.0	●	●	●		●		
**Bandyopadhyay 2011**	7.0			○		○		
**Nicklas 2011**	8.5	●	●	●	●	●	●	●
**Gaudreau 2012**	8.5	●	●		●	●	●	○
**Hjelm 2012**	9.0	●				●	●	
**Jones 2012**	7.5	●			●	●		
**Dasgupta 2013**	9.0	●	●	●		●	●	●
**Lie 2013**	8.5	●	●	●	○	●		○
**Abraham 2014**	8.0		●	○	●	●	●	●
**Morrison 2014**	6.5	●		○	●	●		
**Jones 2015**	8.5	●	●	○		●		●
**O'Dea 2015**	7.5	●	●	●	●			●
**Tang 2015**	8.5	●	●	●		●		
**Lim 2017**	8.0	●	●	●		●		●
**Pennington 2017**	7.5					●		
**Svensson 2017**	8.0	●	●	○	○	●	●	
**Zulfiqar 2017**	8.5	○	●	○	●	●	●	

Large dot: CASP score ≥8.5; medium dot: CASP score 8 (median); small dot: CASP score ≤7.5

Open dots indicate where a study briefly contributes to the theme, or lists the theme

SUPPLEMENTARY TABLE 4: CERQual qualitative evidence profile of recommendations for promoting healthy lifestyles after gestational diabetes

Objective: To systematically synthesise the literature focussing on the views of women with a history of GDM on reducing their risk of developing T2D postpartum

Perspective: Views, experiences and ideas of any women who have had GDM during any previous pregnancy

Included studies: Studies that examine women's postpartum experiences following GDM relating to lifestyle/behaviour, views on T2D risk management and/or experience of a T2D prevention programme

	Review recommendation	Studies directly contributing to the recommendation	Assessment of methodological limitations	Assessment of relevance	Assessment of coherence	Assessment of adequacy	Overall CERQual assessment of confidence	Explanation of CERQual assessment
**Role as mother and priorities**								
**1**	Highlight the benefits to the family of the mother being healthier and role modelling healthy lifestyle to children as the incentive for change, alongside preventing T2D	Dasgupta, Gaudreau, Hjelm, Jones 2015, O'Dea, Svensson, Tang, Razee	Minor concerns: the role of the researcher was poorly considered and implementation of ethical processes was unclear but this was expected to have little impact on answers to this question	No or very minor concerns: many of these studies are directly relevant	Moderate concerns: women in some studies explicitly reported that their children were their motivation for healthy behaviour, while others reported prioritising their children's health; it is unclear whether this should be encouraged and in all women	Minor concerns: women in some studies explicitly reported that their children were their motivation for healthy behaviour, while others reported prioritising their children's health more generally	**Moderate confidence**	Women directly or indirectly reported that their children were their incentive for change; whether it is appropriate for all should be considered
**2**	Include the option of childcare in face-to-face interventions if children are not part of the sessions	Dasgupta, Graco, Lim, O'Dea	Minor concerns: some studies had methodological issues but this was expected to have little impact on answers to this question	No or very minor concerns: these studies are directly relevant	No or very minor concerns: offering childcare is recommended by women in multiple studies; this is also supported by a general concern for children and about childcare	Moderate concerns: relatively few studies contribute to this recommendation and it is not reported in large detail	**Moderate confidence**	Few studies contributed to this recommendation but some directly suggested it and it is supported by general concern about children/ childcare
**Support from family and friends**								
**3**	Promote healthier lifestyles in the wider family (and friends)	Abraham, Dasgupta, Gaudreau, Jones 2015, Lie, Nicklas, Svensson, Zulfiqar	No or very minor concerns: high quality studies contributed to this recommendation	No or very minor concerns: these studies are directly relevant	Moderate concerns: the studies all report that family must eat the same healthier diets (particularly partners) but exercise and the family was less clearly discussed	Moderate concerns: the link between family and diet is well explained but has been extrapolated to include friends and physical activity	**Moderate confidence**	It is clear that women need support for a healthy diet but few studies clearly discussed family and friends exercising
**4**	Encourage the wider family (and friends) to promote healthy lifestyles in mothers and support them practically (such as relieving housework burdens)	Abraham, Dasgupta, Gaudreau, Graco, Jones 2015, Lim, Nicklas, O'Dea, Razee, Svensson, Zulfiqar	Minor concerns: none clearly considered the role of the researcher or implementation of ethics but this was expected to have little impact on answers to this question	No or very minor concerns: many of these studies are directly relevant	No or very minor concerns: these studies specifically reported the crucial role of family and friends in behaviour and none of the studies contradicted the others	No or very minor concerns: studies reported that women directly suggested involving partner; benefited from support; struggled because they lacked support; or said that prioritising their partner prevented healthy behaviour	**High confidence**	Many studies explained the benefits of or need for support for lifestyle change
**5**	Include the family in interventions (eg. information or modules for partners and children)	Abraham, Dasgupta, Nicklas, Zulfiqar	No or very minor concerns: high quality studies contributed to this recommendation	No or very minor concerns: these studies are directly relevant	No or very minor concerns: two studies suggested including family in interventions and the other linked lack of partner attendance at educational sessions (during pregnancy) with lack of postpartum support	Major concerns: only a few studies reported this recommendation, suggesting it as a way of increasing partner support	**Moderate confidence**	Inadequate data reduced our confidence that this recommendation would be useful to postpartum women
**6**	Encourage and facilitate women to exercise with others/a buddy	Dasgupta, Gaudreau, Graco, Nicklas	Minor concerns: high quality studies contributed to this recommendation, although the role of the researcher was poorly considered	No or very minor concerns: these studies are directly relevant	Moderate concerns: some directly suggested having help to find exercise buddies and others reported benefits of socialising while exercising; in addition to the general need for support	Moderate concerns: the studies that directly contributed to this theme did not report the recommendation in much detail	**Moderate confidence**	This recommendation was developed from the general need for support, plus a few studies that specifically addressed it
**Demands of life**								
**7**	Provide guidance about how to buy and prepare healthy, tasty food efficiently	Dasgupta, Evans, Gaudreau, Jones 2012, Jones 2015,	Minor concerns: the role of the researcher was poorly considered and implementation of ethical processes was	Minor concerns: these studies were generally relevant to the review question	Minor concerns: difficulties in meal planning and preparation were frequently reported, and many said they would like more	Minor concerns: this idea was common across studies although the specifics of	**High confidence**	Many women reported the lack of and need for more guidance for having a healthy diet

		Lie, Nicklas, Razee, Zulfiqar	unclear but this was expected to have little impact on answers to this question		help and information (eg. suggested recipe books)	implementing this were less clear		
**8**	Provide guidance about how to exercise around the house and as part of regular daily routines	Abraham, Bandyopadhyay, Dasgupta, Graco, Jones 2015, Lie, Nicklas, Tang, Zulfiqar	Minor concerns: the role of the researcher was poorly considered and implementation of ethical processes was unclear but this was expected to have little impact on answers to this question; there was agreement between higher and lower-quality studies	Minor concerns: these studies were generally relevant to the review question	Moderate concerns: this recommendation was made because time restraints, exhaustion and lack of information were reported to prevent exercise while many reported doing simple exercise in their normal routine; yet others wanted personal trainers or facilities. Some differences may have been due to definitions of exercise	Minor concerns: reasoning behind women's views was well reported and by several studies	**Moderate confidence**	It is clear, and stated, that women need help to increase exercise; however, there is some contradictory suggestions about the best form(s) of exercise to promote and how
**Personal preferences and experiences**								
**9**	Support women to maintain healthy behaviour/diet in challenging situations – eg. social gatherings, breastfeeding, at work (particularly for vulnerable groups)	Bandyopadhyay, Hjelm, Jones 2012, Nicklas, Razee, Zulfiqar	Moderate concerns: the role of the researcher was poorly considered in these studies, which may have had a small effect on women reporting personally challenging situations	Minor concerns: most of these studies were relevant to this review question; both native and migrant populations were studied	Moderate concerns: it is clear that women struggle to maintain healthy diets in challenging situations but none suggested how to help this	Moderate concerns: although this is reported in several studies, this it is relatively vague and broad	**Low confidence**	Certain situations affect women's ability to maintain healthy diets; the best way to address this is unclear
**10**	Highlight the wider benefits of healthier lifestyle (such as reducing stress and weight as well as T2D risk)	Bandyopadhyay, Doran, Gaudreau, Graco, Jones 2012, Jones 2015, Morrison, O'Dea, Razee, Svensson, Tang, Zulfiqar	Minor concerns: these studies had variable quality, particularly around the role of the researcher and implementation of ethical processes, but this was expected to have had a small impact on this recommendation	No or very minor concerns: most of these studies are directly relevant	Minor concerns: many studies reported motivation for healthier lifestyle as T2D prevention or weight loss/body image/ enjoyment, and serval reported both	Minor concerns: this was discussed in some detail by many studies	**High confidence**	Women had identified many benefits of adopting healthier lifestyles that helped them to maintain them (perhaps after their awareness of T2D declined over time)
**Diabetes risk perception and information**								
**11**	Make information, resources and training easily accessible and make interventions available to start immediately after pregnancy (or during pregnancy)	Abraham, Dasgupta, Doran, Evans, Gaudreau, Graco, Hjelm, Jones 2015, Lie, Morrison, Pennington, Razee, Svensson, Zulfiqar	Minor concerns: there was a range of methodological limitations in these studies, but there is agreement with high quality ones	No or very minor concerns: most of these studies are directly relevant	Minor concerns: many reported lacking knowledge about postpartum behaviour; most of these suggested or implied that this should be addressed as early as possible (only Lie reported that an intervention should begin at weaning)	Minor concerns: this recommendation arose from many studies	**High confidence**	This recommendation resulted from many studies that were in agreement, with few exceptions
**12**	Ensure that interventions are culturally appropriate and recommendations allow maintenance of women's identity	Bandyopadhyay, Dasgupta, Gaudreau, Jones 2012, Razee, Zulfiqar	Minor concerns: no studies clearly considered the role of the researcher, which may have had implications for this question, but is unlikely	No or very minor concerns: these studies include migrant or ethnic minority populations; most of the studies included that include such populations report this theme	No or very minor concerns: lack of culturally-specific information was reported as a barrier, presence was a facilitator and some reported women wanting more information	Minor concerns: data is rich in many of the studies	**High confidence**	It was clear that women wanted culturally-relevant interventions and that they were beneficial to those who received it
**13**	Ensure that care providers consider women's attitude towards T2D and advise them on their risk appropriately	Abraham, Bandyopadhyay, Evans, Nicklas, Pennington, Jones 2015, Svensson, Tang, Zulfiqar	Minor concerns: the role of the researcher was poorly considered and implementation of ethical processes was unclear but this was expected to have little impact on answers to this question	No or very minor concerns: most of these studies are directly relevant	Major concerns: this recommendation was based on the finding that women have different attitudes towards T2D (eg. fear or apathy) and some engage or behave differently based on their relationship with clinicians	Major concerns: the studies do not clearly discuss this recommendation	**Low confidence**	This recommendation is a step on from women's attitudes towards behaviour change and their clinician
**14**	Promote a long-term perspective about maintaining healthy lifestyle, with an 'every little helps' approach, rather than 'all or	Bandyopadhyay, Evans, Graco, O'Dea, Zulfiqar	Minor concerns: no studies clearly considered the role of the researcher, which may have had implications for this question, but is unlikely	No or very minor concerns: many of these studies are directly relevant; some were carried out relatively long after pregnancy	Minor concerns: women reported that it was hard to maintain healthy lifestyles, some were daunted by the magnitude of change	Moderate concerns: this was not considered by many studies or in detail; many women appeared to feel that it was just too hard to try (although this	**Moderate confidence**	Paucity of data has reduced our confidence in this recommendation

	nothing', and include the importance of both diet and activity				suggested, and some thought diet was more important than exercise – which should be addressed	was not always explicitly stated by authors)		
**Finances and resources**								
**15**	Provide information about low-cost or money-saving healthy behaviours and resources; interventions should be free	Abraham, Dasgupta, Gaudreau, Hjelm, Nicklas, Svensson, Zulfiqar	Minor concerns: these studies were considered high quality; none clearly considered the role of the researcher or implementation of ethics but this was expected to have little impact on answers to this question	No or very minor concerns: many of these studies are directly relevant	No or very minor concerns: women reported the cost of healthy lifestyle (particularly diet) as a barrier, that they wanted advice on saving money or found that they could save money through healthy lifestyle	Moderate concerns: fewer studies reported this thoroughly but many mentioned the cost of healthy living as a barrier	**High confidence**	There was agreement across studies but this was not reported in detail
**Format of intervention and other**								
**16**	Recommend increasing fruit and vegetable intake, reducing sugar and substituting with healthier ingredients or methods to improve diet	Doran, Evans, Gaudreau, Graco, Hjelm, Lie, Razee	Minor concerns: no studies clearly considered the role of the researcher or implementation of ethics but this was expected to have little impact on answers to this question	Moderate concerns: although the studies are quite directly relevant in terms of study population/setting, the phenomenon of interest is only partially relevant as studies rarely directly asked *what* behaviour change occurred	Minor concerns: this finding is descriptive and none of the studies are contradictory, therefore we have little concern about suggesting it as an approach for others	Minor concerns: women described what changes they had made but not why; no studies reported what women recommended	**Moderate confidence**	Several studies briefly reported women being able to makes these changes
**17**	Recommend flexible exercise such as walking and those performed around the home or with the baby to increase physical activity (rather than attending gyms or classes)	Bandyopadhyay, Dasgupta, Gaudreau, Graco, Hjelm, Jones 2015, Nicklas, O'Dea, Razee, Tang, Zulfiqar	Minor concerns: no studies clearly considered the role of the researcher or implementation of ethics but this was expected to have little impact on answers to this question	Moderate concerns: although the studies are quite directly relevant in terms of study population/setting, the phenomenon of interest is only partially relevant as studies rarely directly asked *what* behaviour change occurred	Minor concerns: this finding is descriptive and only one woman was reported to be concerned about walking; although women in different setting reported different types of exercise, these were all quite flexible	No or very minor concerns: women described why they found walking/flexible exercise the most appropriate to do	**High confidence**	Women across several studies reported how and why they did these types of exercises
**18**	Ensure interventions have web-based components but encourage additional face-to-face contact (they should not depend on women attending sessions)	Dasgupta, Graco, Jones 2015, Lie, Nicklas, O'Dea	Moderate concerns: no studies clearly considered the role of the researcher, which may have had implications when evaluation interventions	No or very minor concerns: evaluating an intervention or studies aiming to inform development of them	Major concerns: many benefits of but barriers to face-to-face contact were reported; there was no agreement in studies regarding the ideal format (online, face-to-face, text messages or telephone call)	Minor concerns: this theme is reported in various levels of richness	**Low confidence**	There was no agreement across studies; this recommendation attempted to consider what women wanted but also what was most practical
**19**	Deliver and promote interventions from recognised/trusted sources (eg. the healthcare provider or a dietitian)	Abraham, Dasgupta, Doran, Gaudreau, Hjelm, Lie, Lim, Lindmark, Nicklas, O'Dea, Pennington, Svensson, Zulfiqar	Minor concerns: a variety of methodical limitations were included but the findings tend to be consistent with high quality studies	No or very minor concerns: many of these studies are directly relevant	Major concerns: these studies report benefits of support offered by various professionals (and some appear to have followed incorrect advice)	Moderate concerns: the findings that this recommendation is based on are rich, but no studies asked who should deliver the intervention	**Low confidence**	Preferred source of the intervention was not discussed; however women reported benefits from their interactions with various professionals
**20**	Promote establishment of systems to monitor progress and accountability (through an intervention or ensure the participant establishes this themselves)	Dasgupta, Gaudreau, Jones 2015, Lim, Nicklas, O'Dea, Tang	Minor concerns: no studies clearly considered the role of the researcher or implementation of ethics but this was expected to have little impact on answers to this question	No or very minor concerns: most included studies were to inform or evaluate interventions	Minor concerns: these studies reported on the need for or benefits of someone to motivate them, and mentioned both formal (eg. clinician) and informal (eg. peer) relationships	Minor concerns: serval studies report this but not very richly; it is in agreement with the general theme of support	**High confidence**	Accountability facilitates behaviour change, but the best way to promote this remains uncertain

Recommendations frequently result from findings within multiple themes but have been presented under the primary contributing theme. Only studies directly contributing to the recommendation have been cited

GDM: gestational diabetes; T2D: type 2 diabetes
